# Comments on “Early Activation of Lung CD8+ T Cells After Immunization with Live *Plasmodium berghei* Malaria Sporozites”

**DOI:** 10.20411/pai.v10i2.851

**Published:** 2025-06-28

**Authors:** Samiha Fatima

**Affiliations:** 1 Sargodha Medical College, Sargodha, Pakistan

## DEAR EDITOR-IN-CHIEF,

The recent study by van Schuijlenburg et al [1] suggests that the lungs may serve as an active and previously underappreciated site of T-cell priming following late-arresting genetically attenuated parasites (LA-GAP) malaria vaccination. Their findings channel traditional liver-focused models and open important avenues for understanding the immunological dynamics of whole-sporozoite vaccines.

Evidence of early and strong activation of lung CD8+ T cells, including elevated production of TNF and Granzyme A, as well as expression of tissue-resident memory T cells (Trms), effector memory T cells (Tem), double negative (DN), and γδ T cells are particularly noteworthy. These findings raise the possibility that lung-resident or lung-primed T cells may contribute meaningfully to protective immunity, either locally or systemically.

While the results stand strongly on their own, they also raise certain questions guiding future research. For instance, the predominance of Granzyme A over Granzyme B or perforin hints at a non-classical cytotoxic profile, potentially involving regulatory or inflammatory roles, as has been described in other infection models [2].

The use of intravenous immunization — which necessarily exposes sporozoites to the lung microenvironment — adds a valuable perspective to pulmonary immune activation.

However, it may be worthwhile to examine how alternative delivery routes such as mosquito bite or intradermal injection affect the immunological arrangement of the lungs, particularly given the differences in antigen-presenting cell recruitment and lymphatic drainage across delivery methods [3, 4].

Another notable observation is the increase in PD-L1+ macrophages in the lungs, which could reflect regulatory signals that may influence T-cell activation. Learning how regulatory signals interact with effector T-cell responses could help clarify the balance between host protection and modulation of inflammation in a lung environment.

Additionally, the study's observation of sex-specific differences in Granzyme A expression and the activation of DN and γδ T-cells highlights the importance of biological variability, which may help explain individual differences in vaccine responsiveness.

These results highlight the importance of lung-mediated immune responses in the prevention of malaria. Future studies that compare delivery routes and dissect the functional roles of lung-primed T-cells could clarify their contribution to protection and inform more targeted vaccine strategies.
